# WRAPPER study: Real‐world effectiveness and tolerability of adjunctive perampanel for people with drug–resistant epilepsy in Hong Kong

**DOI:** 10.1002/epi4.12882

**Published:** 2023-12-26

**Authors:** Charlie C. H. Chan, Ho Wan Leung

**Affiliations:** ^1^ Division of Neurology, Department of Medicine and Therapeutics Prince of Wales Hospital Hong Kong Special Administrative Regions China; ^2^ Present address: Department of Medicine and Therapeutics Prince of Wales Hospital Hong Kong Special Administrative Regions China

**Keywords:** Asian, drug resistant epilepsy, Hong Kong, perampanel

## Abstract

**Objective:**

The Prince of Wales Hospital (PWH) Real‐world Analysis of People with Drug‐Resistant Epilepsy (DRE) on PERampanel (WRAPPER) study assessed effectiveness and tolerability of adjunctive perampanel in people with DRE attending PWH.

**Methods:**

This was a prospective single‐center real‐world observational study involving 70 people with DRE between July 2016 and June 2021. A post hoc analysis after the initial study period of 16 weeks assessed outcomes for an extended period up to 52 weeks.

**Results:**

After 16 weeks, median dose of perampanel was 2 mg (IQR 24 mg). 50% responder rates were 40.0%, 41.5%, and 48.7% at 16, 26, and 52 weeks. Seizure freedom was 12.9%, 20.7%, and 25.6% at 16, 26, and 52 weeks. Monthly seizure frequency reduced from 3.0 (IQR 3.0–6.6) at baseline to 2.0 (IQR 2.0–6.0, *p* = 0.005) at 16 weeks; 2.0 (IQR 2.0–5.0, *p* = 0.01) at 26 weeks; and 2.0 (IQR 0.0–4.0, *p* = 0.018) at 52 weeks. Older age predicted 50% responders (OR 1.08, 95% CI 1.01–1.14, *p* = 0.048).

At 16 weeks, 51.4% (36/70) had treatment‐emergent adverse effects (TEAEs). Most common was seizure exacerbation at 35.7% (25/70) followed by fatigue at 15.7% (11/70). NPI‐12 and ZBI scores indicated no increase in neuropsychiatric symptoms on perampanel.

**Significance:**

Low‐dose 2–4 mg adjunctive perampanel for people with DRE conferred appreciable improvements in seizure reduction without significant neuropsychiatric adverse effects in the real‐world setting at a tertiary center in Hong Kong and had better antiseizure effect with advancing age.

**Plain Language Summary:**

This real‐world study from Hong Kong found low‐dose perampanel was effective and tolerable for people with drug‐resistant epilepsy. Furthermore, perampanel was also potentially more effective with advancing age.

**Key points**

- Low‐dose adjunctive perampanel at 2–4 mg for people with DRE conferred appreciable improvements in seizure reduction in this Asian cohort.
- Adjunctive perampanel appears to have better antiseizure effect with advancing age.
- Adjunctive perampanel achieved seizure freedom in up to 25% within this cohort of people with DRE.
- Real‐world data from this cohort indicate neuropsychiatric side effects of perampanel were minimal.
- Furthermore, studies to assess effectiveness of low‐dose perampanel in PWE of advancing age and Asian ethnicity appear promising.

## 1 INTRODUCTION

Drug‐resistant epilepsy (DRE) is defined as failure to achieve seizure freedom after trials of 2 or more appropriately chosen, adequately dosed, and properly taken antiseizure medication (ASM) schedules whether as monotherapy or in combination.^1^ This is based on the finding that if seizure freedom cannot be achieved with the initial 2 ASMs, likelihood of success is progressively lower with subsequent ASMs.^2^ Figures from the International Leagues Against Epilepsy (ILAE) reports 1‐year seizure freedom rates drop from 45.7% on 1 ASM, to 28% on 2 ASMs, 23.6% on 3 ASMs, 15% on 4 ASMs, 14.1% on 5 ASMs, 14% on 6 ASMs, and 6.7% on 7 ASMs.^3^ DRE is also not uncommon; nearly a third of people with epilepsy (PWE) have DRE.^4^ This is a significant cost to society as these people have poorer quality of life, family function, and social support compared to other chronic conditions and are at increased risk for sudden unexpected death in epilepsy (SUDEP).^5^, ^6^

Despite development of numerous ASMs, evidence indicates no significant improvement in seizure control. A longitudinal cohort of newly diagnosed epilepsy from 1982 to 2012 in Scotland found seizure‐free rate was 64.0% in the early 2000s, 68.2% in 2008, and 63.7% in 2014 despite increasing use of new ASMs.^7^ Among these new ASMs is perampanel. The aforementioned cohort only had less than a year of perampanel data before stopping recruitment. With its novel mode of action, there is hope that perampanel can make a difference for DRE.

Perampanel is the first orally active non‐competitive AMPA receptor antagonist approved for adjunctive therapy in epilepsy in 2012 in the United States and European Union.^8^ Efficacy, safety, and tolerability of perampanel were established in four large multinational, multicenter, double‐blinded placebo‐controlled randomized phase 3 trials: 304, 305, 306, and 335 trials.

304 demonstrated that in participants ≥12 years old with refractory focal seizures with or without secondary generalization, adjunctive perampanel 8–12 mg was safe and tolerable.^9^

305 demonstrated that in participants ≥12 years old with refractory focal seizures with or without secondary generalization, adjunctive perampanel 8–12 mg was safe, tolerable, and statistically significantly improved 50% responder rates.^10^

306 demonstrated that in participants ≥12 years old with refractory focal seizures with or without secondary generalization, adjunctive perampanel 4–12 mg effectively reduced seizure frequency alongside a favorable tolerability profile.^11^

Pooled analysis of 304, 305, and 306 trials reaffirmed these findings of significant improvements in seizure control on perampanel 4–12 mg. Interestingly, responder rates were lower in participants receiving concomitant carbamazepine than in those receiving concomitant non‐enzyme‐inducing ASMs.^12^

307 was an extension study of 304 and 305 participants up to 3 years. 307 showed perampanel 12 mg conferred sustained reduction in seizure frequency with favorable tolerability profile beyond 1 year.^13^

335 demonstrated that in Asian participants ≥12 years old with refractory focal seizures with or without secondary generalization, adjunctive perampanel 8–12 mg reduced seizure frequency.^14^

Of interest, in 335 perampanel, 4 mg had no statistically significant difference in seizure reduction when compared to placebo, whereas in 306 perampanel, 4 mg did demonstrate a statistically significant difference in seizure reduction compared to placebo. The authors of 335 acknowledged this difference may be due to the 335 cohort being more drug refractory when compared to 306 as evidenced by a higher proportion of participants taking three concomitant ASMs at baseline.^14^

Data from these four trials underwent pooled analysis comparing efficacy, safety, and tolerability of perampanel in Asian versus non‐Asian participants with epilepsy. In summary, there were no differences between Asian and non‐Asian populations.^15^

Local data for perampanel in Hong Kong were previously reported in a retrospective study by Chang et al. The reported 50% responder rate was 32.1%, and seizure freedom rate 13.2%, with a treatment‐emergent adverse event (TEAE) withdrawal rate 28.3%.^16^

People with DRE eagerly await better treatment options. Findings from the previously quoted randomized control trials may not be entirely generalizable as study participants must meet exclusive inclusion criteria and follow strict study protocols. Real‐world data from a heterogeneous cohort in real‐world settings may generate evidence better applicable to people with DRE seen in clinics and wards. The objective of this study was to assess real‐world effectiveness and tolerability of perampanel in people with DRE in Hong Kong.

## 2 METHODS

The Prince of Wales Hospital (PWH) Real‐world Analysis of People with Drug‐Resistant Epilepsy on PERampanel (WRAPPER) study was an observational prospective single‐center study conducted at the Prince of Wales Hospital (PWH) in Shatin, Hong Kong, in accordance with the Declaration of Helsinki and ICH‐E6 Guideline for Good Clinical Practice; Joint Chinese University of Hong Kong‐New Territories East Cluster Clinical Research Ethics Committee (CUHK‐NTEC CREC) Reference Number 2016.314. All participants provided written informed consent.

Eligible people with DRE were recruited and initiated on adjunctive perampanel in the study period of 16 weeks. Subject to the investigator's clinical assessment, perampanel could be increased or reduced. Participants could opt to stop the medication before 16 weeks.

The primary effectiveness endpoint was 50% responder rate at enrollment (0 weeks) and at the end of the study period (16 weeks). The secondary effectiveness endpoint was 25% responder rate, seizure freedom, and changes in median monthly seizure frequency. Extended follow‐up beyond the study period at 26 and 52 weeks was traced via computerized records to assess the performance of perampanel over a longer duration.

Perampanel has been reported to have neuropsychiatric effects; as such, the 12‐item neuropsychiatric inventory (NPI)‐12 questionnaire and 22‐item Zarit Burden Interview (ZBI) were administered upon recruitment and at the end of the study period. Tolerability endpoints included changes in NPI‐12 scores, change in ZBI scores, development of perampanel‐related TEAEs, and retention rates.

## 3 STUDY POPULATION

All people with DRE attending Prince of Wales Hospital from July 2016 to June 2021 were prospectively screened for inclusion.

### 3.1 Inclusion criteria

1. ≥12 years old
2. Epilepsy type: focal epilepsy
3. Baseline seizure frequency ≥2 per month in preceding 8 weeks before recruitment
4. No seizure‐free period longer than 21 days during 8 weeks before recruitment

No restriction was specified on the number of concomitants EIASMs to reflect real‐world practice.

### 3.2 Exclusion criteria

1. Epilepsy type: generalized epilepsy, combined generalized, and focal epilepsy
2. Severe renal impairment defined as calculated creatinine clearance ≤50 mL/min
3. Severe liver impairment defined as ALT ≥3 times upper limit normal
4. Significant psychiatric comorbidity before initiation of perampanel
5. Progressive neurodegenerative conditions
6. Active malignancy
7. History of hematological diseases
8. Corrected QT interval ≥ 450 ms on ECG
9. Substance use disorder
10. Pregnancy
11. Breastfeeding

Data were entered into the computerized Clinical Management System (CMS) under the Hong Kong Hospital Authority (HA) at 0 and 16 weeks. Thereafter, participants continued follow‐up as indicated in routine practice in the neurology clinic at PWH. Baseline demographic and seizure frequencies were cross‐checked and consolidated into a database by an epilepsy nurse.

The following data were collected: demographics; epilepsy type, and etiology of epilepsy as per the updated ILAE Classification of the Epilepsies 2017^17^; age of onset; duration of epilepsy; psychiatric comorbidities; previous and concomitant ASMs; seizure frequency. Clinical diaries were used to record seizures, and this information was transcribed into the CMS.

Statistical analyses were performed using SPSS Statistics, version 27.0 (IBM Corporation, Armonk, NY, USA). The threshold of significance was *p* < 0.05. The Benjamini–Hochberg (BH) procedure was applied to control for a false discovery rate after multiple comparisons. Descriptive analysis of categorical and continuous variables collected was performed.

Categorical variables were expressed as frequencies and percentages. The chi‐square test was applied for variables with at least 80% of cells having an expected frequency of ≥5. Fisher's exact test was used for variables with 80% of cells having an expected frequency <5.

Continuous variables satisfying Kolmogorov–Smirnov test for normality were analyzed by their mean, standard deviation, and *t*‐test. For non‐parametric continuous variables, median and interquartile ranges were reported instead. The Mann–Whitney *U* test was applied for non‐parametric independent samples. A paired Wilcoxon signed rank test was applied for non‐parametric dependent samples.

Exploratory analysis of baseline variables to predict 50% responders, seizure freedom, and retention on perampanel was performed. All comparisons reaching threshold of *p* < 0.05 on two‐sided tests were pre‐selected as independent variables for a multivariate binary logistic regression.

Retention on perampanel up to 52 weeks from initiation was assessed via Kaplan–Meier calculations.

## 4 RESULTS

Data for 70 participants were collected. Participants' characteristics and baseline ASM regimens are summarized in Table 1. The oldest participant was 64 years old. Perampanel was initiated at 2mg once daily, by 16 weeks the median dose of perampanel was 2 mg once daily (IQR 2‐4 mg).

**TABLE 1:** Clinical characteristics of the WRAPPER cohort.

Clinical characteristics	Value
Age, years, median (IQR)	38.0 (31.8–46.0)
Age at epilepsy onset, years, median (IQR)	15.5 (7.8–28.5)
Duration of epilepsy, years, median (IQR)	20.0 (10.0–29.0)
Sex, female, *n* (%)	45.0 (64.2)
Epilepsy etiology, *n* (%)	
Structural	39.0 (55.7)
Genetic	3.0 (4.3)
Infectious	11.0 (15.7)
Immune	3.0 (4.3)
Metabolic	1.0 (1.4)
Cryptogenic	13 (18.6)
Type of seizures at baseline	
Focal preserved awareness seizure	17.0 (24.3)
Focal impaired awareness seizure	69.0 (98.5)
Focal to bilateral tonic clonic seizure	46.0 (65.7)
Seizure Frequency at Baseline, median (IQR)	3.0 (2.0–6.95)
Number of previous ASMs, median (IQR)	5.0 (2–8)
Number of concomitant ASMs, median (IQR)	2.0 (2–3)
1, *n* (%)	12.0 (17.1)
2, *n* (%)	23.0 (32.9)
3, *n* (%)	21.0 (30.0)
4, *n* (%)	11.0 (15.8)
5, *n* (%)	2.0 (2.9)
Concomitant enzyme inducing ASMs (EIASM), *n* (%)	48.0 (68.6)
Carbamazepine	24.0 (34.3)
Oxcarbazepine	14.0 (20.0)
Phenobarbitone	5.0 (7.1)
Phenytoin	8.0 (11.4)
Sodium channel blocking ASMs, *n* (%)	65 (92.9)
Carbamazepine	24.0 (34.3)
Lacosamide	20.0 (28.6)
Lamotrigine	19.0 (27.1)
Oxcarbazepine	14.0 (20)
Phenytoin	8.0 (11.4)

Abbreviations: ASM, antiseizure medication; IQR, interquartile range.

### 4.1 Effectiveness of perampanel

At all time points, there were statistically significant reductions in median monthly seizure frequency. Upon recruitment, median monthly seizure frequency was 3.0 (IQR 3.0–6.6); at 16 weeks, 2.0 (IQR 2.0–6.0, *p* = 0.005); at 26 weeks, 2.0 (IQR 2.0–5.0, *p* = 0.01); at 52 weeks, 2.0 (IQR 0.0–4.0, *p* = 0.018). Percentage change in seizure frequency at 16, 26, and 52 weeks was 41.3% (IQR −4.9 to 83.4), 24.2% (IQR −14.3 to 75.0), and 44.4% (IQR −33.3 to 90.0), respectively.

At 16 weeks, 40.0% (28/70) achieved 50% responder rate; 53.0% (37/70) achieved 25% responder rate; and 12.9% (9/70) achieved seizure freedom. Seizure exacerbation was reported in 35.7% (25/70).

At 26 weeks, 41.5% (22/53) achieved 50% responder rate, 49.1% (26/53) achieved 25% responder rate; 20.7 (11/53) achieved seizure freedom. Seizure exacerbation was reported in 28.3% (15/53).

At 52 weeks, 48.7% (19/39) achieved 50% responder rate, 56.4% (22/39) achieved 25% responder rate; and 25.6% (10/39) achieved seizure freedom. Seizure exacerbation was reported in 33.3% (13/39).

### 4.2 Exploratory analysis of baseline characteristics

Exploratory analysis of baseline characteristics associated with higher likelihood of 50% responder rate, 25% responder rate, and seizure freedom was done at each time point.

At 16 weeks, the proportion of 50% responders was paradoxically statistically significantly higher in participants taking carbamazepine (OR 3.13, 95% CI 0.99–9.85, *p* = 0.046) and on EIASM (OR 3.20 95% CI 1.15–8.93, *p* = 0.024). No other participant or disease‐related factors correlated with better or poorer effectiveness. At 16 weeks, binary logistic regression found no statistically significant variables associated with likelihood of achieving 50% responder rate.

**FIGURE 1:**
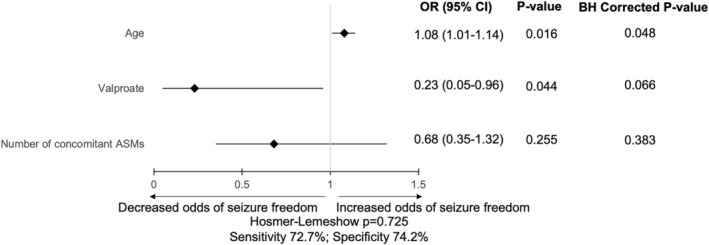
Multivariate analysis of relationships between baseline characteristics and 50% responder rate.

At 26 weeks, the proportion of 50% responders was statistically significantly higher in older participants (*p* = 0.010) and participants who had a fewer number of concomitant ASMs (*p* = 0.021) but lower in participants taking concomitant valproate (OR 0.28, 95% CI 0.08–0.94, *p* = 0.034). No disease‐related factors correlated with effectiveness outcomes. These comparisons reaching *p* < 0.05 were entered into binary logistic regression to determine ability to predict 50% responders. Results of this binary logistic regression are in Table 2 and Figure 1. After Benjamini–Hochberg correction for multiple comparisons, age remained the only statistically significant variable associated with likelihood of achieving 50% responder rate.

**TABLE 2:** Binary logistic regression of factors associated with 50% responders at 26 weeks.

Independent variable	Coefficient	Standard error	OR (95% CI)	*p*‐Value	BH *p*‐Value
Age	0.074	0.031	1.08 (1.01–1.14)	**0.016**	**0.048**
VPA	−1.490	0.738	0.23 (0.05–0.96)	**0.044**	0.066
Number of concomitant ASMs	−0.388	0.341	0.68 (0.35–1.32)	0.255	0.383

*Note*: Sensitivity 72.7%; Specificity 74.2%; Percentage accuracy in classification (PAC) 73.6%. Bold indicates to highlight these *p*‐values remained statistically significant after BH correction.

Abbreviations: ASM, antiseizure medication; BH, Benjamini–Hochberg corrected; CI, confidence interval; OR, odds ratio; VPA, valproate.

At 52 weeks, there were no statistically significant associations between participant, disease, or medication‐related factors relating to effectiveness outcomes.

In summary, age was the only factor demonstrating statistically significant association with 50% responder rate at any time point in this cohort. There were no medication or disease‐related factors including immune etiologies of epilepsy or vascular.

### 4.3 Tolerability

**FIGURE 2:**
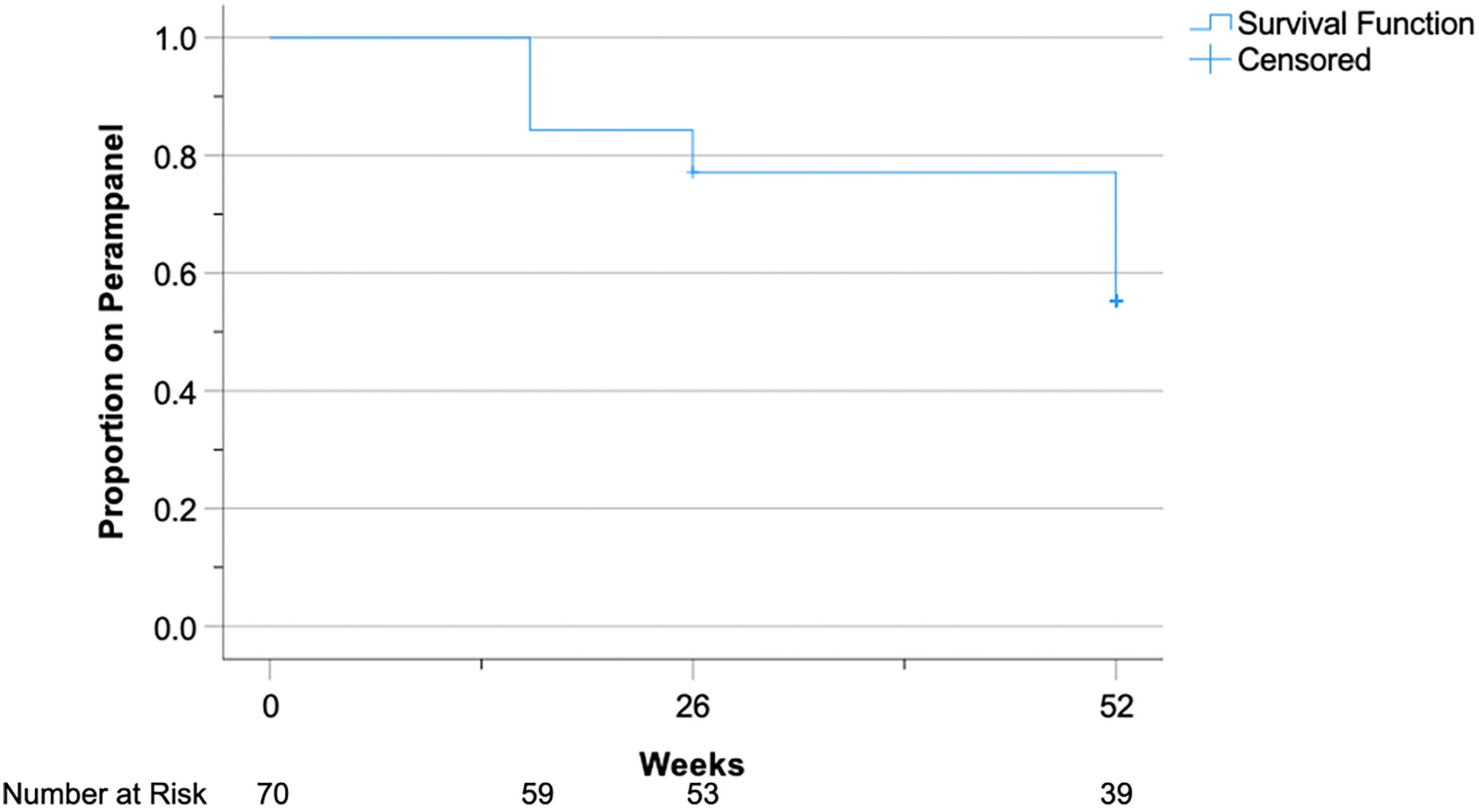
Kaplan–Meier curve for retention on perampanel treatment over 52 weeks.

59 participants completed 16 weeks of perampanel. Retention on perampanel at 16, 26, and 52 weeks was 84.2% (59/70), 89% (53/59), and 73.6% (39/53), respectively. Mean retention time on perampanel treatment was 44.5 weeks (SD 1.7, 95% CI 41.2–47.8). These findings are summarized graphically in Figure 2.

At 16 weeks, 51.4% (36/70) had reported treatment‐emergent adverse effects (TEAE). TEAEs reported by participants while on perampanel in the current cohort are summarized in Appendix S1. Descriptive analyses found seizure exacerbation as the only adverse effect predicting withdrawal from perampanel. All other recorded adverse effects were not statistically significantly associated with perampanel withdrawal after Benjamini–Hochberg correction.

12‐item neuropsychiatric inventory (NPI‐12) data were available for 59 cases after 16 weeks. Upon recruitment, 51 cases (86.4%) reported having psychiatric complaints. After 16 weeks of perampanel, 50 cases (84.7%) reported having psychiatric complaints after perampanel. NPI‐12 scores at 0 weeks and 16 weeks were compared, and statistically significant differences are summarized in Table 3. Depression/dysphoria was the only domain with statistically significant change. Despite data being not normally distributed, the means and standard deviations have been shown to better demonstrate the change between initiation and 16 weeks.

**TABLE 3:** Comparison of neuropsychiatric inventory scores at 0 and 16 weeks with statistical significance.

Neuropsychiatric inventory measure	Week 0	Week 16	*p*‐Value	BH *p*‐Value
D: Depression/Dysphoria, *n* (%)	22 (37.3)	11 (18.7)		
Frequency, mean ± SD	0.64 ± 0.97	0.29 ± 0.70	0.001	**0.025**
Severity, mean ± SD	0.57 ± 0.84	0.28 ± 0.64	0.002	**0.033**
Frequency × severity, mean ± SD	1.07 ± 2.10	0.51 ± 1.56	0.001	**0.025**
Caregiver distress, mean ± SD	0.64 ± 1.120	0.34 ± 0.91	0.004	**0.049**
H: Disinhibition, *n* (%)	0 (0.0)	4 (6.8)		
Frequency, mean ± SD	0 ± 0	0.07 ± 0.26	0.046	0.250
Severity, mean ± SD	0 ± 0	0.07 ± 0.26	0.046	0.250
Frequency × severity, mean ± SD	0 ± 0	0.07 ± 0.26	0.046	0.250
Caregiver distress, mean ± SD	0 ± 0	0.05 ± 0.29	0.180	0.519
I: Irritability/Lability, *n* (%)	14 (23.7)	21 (35.6)		
Frequency, mean ± SD	0.29 ± 0.56	0.63 ± 1.04	0.015	0.147
Severity, mean ± SD	0.29 ± 0.56	0.47 ± 0.68	0.088	0.359
Frequency × severity, mean ± SD	0.36 ± 0.76	0.88 ± 1.70	0.072	0.321
Caregiver distress, mean ± SD	0.37 ± 0.87	0.52 ± 0.94	0.218	0.511

*Note*: Bold indicates to highlight these *p*‐values remained statistically significant after BH correction.

Abbreviations: BH, Benjamini–Hochberg corrected; SD, standard deviation.

For disinhibition and irritability/lability, there was an increase after 16 weeks, but this was not statistically significant. For other NPI‐12 domains, there was no statistically significant change after administration of 16 weeks of perampanel. A subgroup analysis of changes in NPI‐12 scores for participants on concomitant levetiracetam was carried out and found no statistically significant changes across all NPI‐12 domains before or after perampanel in these participants. In summary, comparing NPI‐12 scores at recruitment and at 16 weeks, perampanel had not induced any significant neuropsychiatric adverse effects for both participants and their caregivers.

ZBI scores at 0 weeks and 16 weeks were 11 (0–42) and 8 (0–38), respectively. Comparison by the paired sample Wilcoxon signed rank test found no statistically significant difference (*p* = 0.166).

## 5 DISCUSSION

Results of WRAPPER demonstrates in real‐world setting, perampanel was effective and well tolerated for the treatment of people with DRE in Hong Kong. At 16 weeks, 50% responder rate was 40.0%, and 12.9% achieved seizure freedom, with a retention rate of 81.4%.

There was statistically significant reduction in median number of seizures from 3.0 (IQR 3.0–6.6) to 2.0 (IQR 2.0–6.0, *p* = 0.005) translating to 41.3% reduction in seizure frequency. Seizure reduction from 3.0 to 2.0 may not appear significant at first glance, but for any seizure event prevented, the possibility of resultant injury or hospitalization is averted, and when translated to a yearly seizure rate, the seizure reduction becomes considerable. These measures of effectiveness found in WRAPRER are comparable to findings of other real‐world perampanel studies as outlined in Table 4.

**TABLE 4:** Overview comparison of real‐world perampanel studies.

	WRAPPER	PERMIT^18^	Japanese^18^	FYDATA^19^	Australian DRE^20^	Chinese^19^
No. of cases	70	5193 (Europe, Asia, Australia, North America)	3808	464 (Spain)	387	186 (Huashan)
Type of epilepsy	Focal DRE	Any epilepsy	Focal epilepsy	Focal epilepsy	Any DRE	Focal epilepsy
Mean/median dose of perampanel	2 mg IQR 2–4 mg 16 weeks	2.4 mg at baseline 6.3 mg at last visit	3.7 ± 1.9 mg overall 3.8 ± 2.0 mg <65 years 3.0 ± 1.5 mg ≥65 years	5.3 at 3 months 6.9 at 12 months	8 mg at 1 year	4.3 ± 2.7 mg at 1 year 2 mg most common dose 52.8%
50% Responder	40.0–48.7%	50.0–58.3%	60.1% <65 years 89% ≥65 years	40.2–47.3%	21.7–38.0%	59.1–66.6%
Seizure Freedom	12.9–25.6%	20.5–23.2%	38.0% <65 years 78.0% ≥65 years	7.5–11.5%	9.0–16.3%	26.8–39.5%
Predictors of efficacy	Older age	Older age at epilepsy onset, genetic etiology of epilepsy, absent psychiatric comorbidity, lower number of previous ASMs, lower number of concomitant ASMs	Older age ≥65 years, baseline seizure type GTC, genetic/vascular/brain tumour etiologies of epilepsy	Older age ≥65 years, vascular etiology of epilepsy, lower number of previous ASMs	Older age, older age at epilepsy onset	Sleep related epilepsy, younger PWE with BECTS, fewer concomitant ASMs
Older age effectiveness	1.08 (1.01–1.14)	(1.01–1.02)	2.02 (1.45–2.82)	3.19 (1.04–9.69)	1.03 (1.01–1.06)	N/A
Odds ratio (95% CI)	*p* = 0.048	*p* < 0.001	*p* < 0.001	*p* = 0.040	*p* = 0.006	

Abbreviations: ASM, antiseizure medication; BECTS, benign epilepsy with centrotemporal spikes; DRE, drug resistant epilepsy; FYDATA: The FYDATA (Follow‐up of 1 year data of patients on perampanel) study; IQR, interquartile range; mg, milligram; PERMIT: PERampanel pooled analysis of effectiveness and tolerability (PERMIT) study; PWE, people with epilepsy; WRAPPER: Prince of Wales Hospital (PWH) Real‐world Analysis of People with Drug Resistant Epilepsy (DRE) on PERampanel (WRAPPER) study.

Findings in WRAPPER are comparable to findings of PERMIT, the largest pooled analysis of real‐world data regarding PWE with epilepsy treated with perampanel.^22^ PERMIT included PWE from a wide spectrum, not only DRE. Keeping this in mind, the lower 50% responder rate of 40.0% in WRAPPER versus that of 58.3% reported in PERMIT is reasonable. Similarly, seizure freedom of 12.9%–25.6% in WRAPPER versus that of 23.2% reported in PERMIT is likely attributable to a more drug refractory cohort. This is reflected by differences in ASM regimens; in WRAPPER, the number of previous ASMs was 5.0 (2–8) versus 4.0 (0–19) in PERMIT.

Of note, median dose of perampanel in WRAPPER was only 2 mg (IQR 2–4 mg) after 16 weeks compared to 6.3 mg at last visit in PERMIT; but even with this lower dose of perampanel, statistically significant reduction in seizure frequency was observed in WRAPPER. It may be argued that WRAPPER has no comparison arm such that improvements in seizure frequency can still be attributable to placebo. Moreover, with reference to 306, perampanel 2 mg had not demonstrated any statistical difference from placebo for seizure reduction. However, if comparison is made to real‐world cohorts from Japan and China, the potential effectiveness of this lower dose of perampanel in a real‐world setting becomes more interesting.

From Table 4, the three exclusively Asian trials had lower median/mean doses of perampanel. A Huashan cohort reported a mean dose of perampanel 4.3 mg ± 2.7 mg at 1 year with perampanel 2 mg being the most commonly prescribed dose. From a Japanese cohort of 3808 participants, 50% responder rates were 60.1% for those <65% and 89.0% for those ≥65 years. Furthermore, breakdown of perampanel dosage by age groups in the Japanese study reveals the older group had a lower average dose of 3.0 mg in ≥65 years versus 3.8 mg in <65 years. These real‐world findings suggest perampanel 2 mg in the Asian population, especially elderly, may be an effective dose contrary to the findings of 306 and 335. A closer look at the participants included in 304–306 will reveal that elderly participants ≥65 years only made up 2.1% (28/1335) of the study population; thus, the original phase 3 trials for perampanel only had limited evidence for its use in elderly PWE.

Indeed, further perusal of multiple real‐world studies, including those beyond Asia, have findings that older age seems to be a predictor for better seizure control with perampanel. FYDATA, a Spanish cohort, and an Australian cohort of people with DRE all found older age was an independent variable for predicting better response to perampanel on multivariable binary logistic regression. However, it should be noted the predictive effect of age was not always substantial despite being statistically significant.

Undeniably, perampanel use in elderly Asian PWE has been encouraging. This trend of better seizure control in elderly Asians with perampanel has also been observed by epilepsy experts across Asia such that an expert opinion article was published in 2022.^23^

Regarding tolerability, mean retention time in WRAPPER was 44.5 weeks (SD 1.7, 95% CI 41.2–47.8) comparable to that of PERMIT at 43.2 weeks (95% CI 42.4–43.6). Seizure exacerbation was the most common adverse effect at 35.7% (25/70) at 16 weeks. PERMIT reported a lower proportion of seizure exacerbations ranging from 6.6% to 10.1% at various time points. Indeed, the comparatively higher rates of seizure exacerbation observed in this cohort are concerning. Yet, further statistical analyses did not reveal any specific participants, medication, or disease‐related factors that predicted seizure exacerbation at all time points in WRAPPER. Upon further review of the specific cases that had been coded as having seizure exacerbations, it was noted some had reduction in seizure frequency but felt the severity of their seizures had increased; others reported an increase in seizure frequency but a reduction in seizure severity. As a real‐world study, whether participants themselves felt perampanel had been effective and tolerable was also an important assessment to be recorded. As such, for all participants who felt that perampanel had worsened their seizure control regardless of improvements in frequency or severity, such cases were classified as having seizure exacerbation. This likely resulted in a larger number of those who felt perampanel was not tolerable for them but also allowed effectiveness data to be analyzed with an intention to treat like approach. In the future, to better reflect such patient‐reported outcomes, a standardized instrument such as the Personal Impact of Epilepsy Scale (PIES) may be utilized instead.^24^

Following seizure exacerbation, fatigue was the next most common adverse effect in WRAPPER at 15.7% (11/70). In comparison, PERMIT reported fatigue at 3.2% only. A larger cohort of participants with all types of epilepsy, not only DRE, would probably be needed assess the potential factors for this difference found.

Neuropsychiatric adverse effects have been commonly reported in clinical trials of perampanel. Indeed, in PERMIT, a considerable proportion of PWE had psychiatric adverse effects, up to 21.0%; moreover, 9.6% discontinued perampanel due to these psychiatric adverse effects. For reference, the proportion of participants in PERMIT with psychiatric comorbidities was 51.2% In WRAPPER, at recruitment, 86.4% had psychiatric complaints compared to 84.7% after 16 weeks of perampanel. This significant difference in prevalence of psychiatric complaints in Hong Kong to figures reported in PERMIT may be attributable to the administration of NPI‐12. One may argue this is not reflective of real‐world clinical practice, but upon closer analysis of the difference in NPI‐12 scores before and after perampanel, even in the presence of a cohort with a higher prevalence of psychiatric complaints, perampanel use did not statistically significantly increase psychiatric adverse effects. Specific subgroup analysis for participants in WRAPPER taking concomitant levetiracetam and perampanel also did not reveal statistically significant changes in NPI‐12 and ZBI scores.

In WRAPPER, only depression/dysphoria had statistically significant reduction after taking perampanel. There were increases in irritability/lability, but these did not reach the threshold of statistical significance. Overall, findings from WRAPPER suggest psychiatric complaints are very common in participants with DRE, and the administration of perampanel did not significantly worsen these psychiatric comorbidities.

The strengths of WRAPPER include its prospective design and provision of real‐world data and evidence from a cohort of people with DRE in Hong Kong. The limitations of WRAPPER include its single‐center design, small sample size, alongside only relatively brief duration of follow‐up. At the time of initial recruitment back in July 2016, perampanel had not yet been stocked by the Hospital Authority Drug Formulary and as a result may have stifled the number of participants that could continue perampanel beyond 16 weeks. As a result of this time limit, there were participants who were unable to tolerate a quicker up titration of perampanel to the suggested dose from the RCTs to above 4 mg. As such, there remains a possibility the effectiveness outcomes could be even better if the median dose for this cohort could be increased.

At the time of writing, perampanel has now been purchased by the Hospital Authority; it would be worthwhile now to track down and follow up all PWE under care by the Hospital Authority on perampanel for a longer duration to gather a larger body of evidence. Inclusion of a wider spectrum of PWE including the elderly, those with status epilepticus, and even those who have started perampanel as monotherapy would be of specific interest to help guide real‐world practice.

In summary, real‐world findings of WRAPPER demonstrate perampanel is a safe and effective adjunctive ASM for people with DRE in Hong Kong. Previously highlighted concerns of perampanel‐related neuropsychiatric adverse effects in clinical trials were not observed in real‐world setting. Even in a DRE cohort, up to a quarter of participants achieved seizure freedom after addition of adjunctive perampanel. Low‐dose perampanel 2–4 mg seemed to already provide improvements in seizure prophylaxis, a finding also seen in other real‐world Asian cohorts. Concomitant EIASMs did not significantly affect the effectiveness of adjunctive perampanel in WRAPPER. Older age may be a predictor for 50% responders to adjunctive perampanel in people with DRE, a trend observed in multiple real‐world cohorts worldwide. A future area of interest may be real‐world effectiveness and tolerability of low‐dose perampanel 2–4 mg in elderly Asian PWE for seizure prophylaxis given the trends found in WRAPPER and corroborated independently by multiple groups internationally.

## AUTHOR CONTRIBUTIONS

Charlie C. H. Chan designed the analysis, collected the data, performed the analysis, and wrote this manuscript. Howan Leung conceived and designed the analysis, and collected the data.

## FUNDING INFORMATION

Eisai Hong Kong funded the post‐marketing Phase IV study.

## CONFLICT OF INTEREST STATEMENT

None of the authors has any conflict of interest to disclose. We confirm that we have read the journal's position on issues involved in ethical publication and affirm that this report is consistent with those guidelines.

## ETHICS STATEMENT

This study was approved by the joint Chinese University of Hong Kong‐New Territories East Cluster Clinical Research Ethics Committee (CUHK‐NTEC CREC), Reference Number 2016.314.

## Supporting information


Appendix S1.

## Data Availability

The data that support the findings of this study are available from the corresponding author, upon reasonable request.
