# Supplementary material for: WRAPPER study: Real‐world effectiveness and tolerability of adjunctive perampanel for people with drug–resistant epilepsy in Hong Kong
Source: Epilepsia Open. 2023 Dec 26;9(1):345–54. doi: 10.1002/epi4.12882 (PMC10839329; doi:10.1002/epi4.12882)
Supplement: Supplementary file 1 — Appendix S1. [file EPI4-9-345-s001.docx]

Appendix A: Treatment Emergent Adverse Effects from Perampanel

| Treatment Emergent Adverse Effects (TEAE) | Perampanel Withdrawal  *n*=13, 18.5% | Perampanel Continuation  *n*=57, 81.4% | OR (95% CI) | P-value | BH P-value |
| --- | --- | --- | --- | --- | --- |
| Seizure exacerbation (25), *n* (%) | 11 (84.6) | 14 (24.6) | 16.89 (3.33-85.61) | **0.000098** | **0.002** |
| Fatigue (11), *n* (%) | 1 (50.0) | 10 (17.5) | 4.70 (0.27-81.63) | 0.341 | 1.000 |
| Memory impairment (7), *n* (%) | 2 (100.0) | 5 (8.8) | 1.40 (0.8802.24) | **0.012** | 0.096 |
| Dizziness (6), *n* (%) | 0 (0.0) | 5 (10.5) | 0.96 (0.91-1.02) | 1.000 | 1.000 |
| Behavioural problem (5), *n* (%) | 0 (0.0) | 5 (8.8) | 0.96 (0.91-1.02) | 1.000 | 1.000 |
| Weight gain (4), *n* (%) | 1 (50.0) | 3 (5.3) | 18.00 (0.89-363.63) | 0.132 | 1.000 |
| Headache (3), *n* (%) | 0 (0.0) | 3 (5.3) | 0.96 (0.92-1.01) | 1.000 | 1.000 |
| Rash (2), *n* (%) | 0 (0.0) | 2 (3.5) | 0.97 (0.92-1.01) | 1.000 | 1.000 |
| Menorrhagia (2), *n* (%) | 0 (0.0) | 2 (3.5) | 0.97 (0.92-1.01) | 1.000 | 1.000 |
| Tremor (1), *n* (%) | 0 (0.0) | 1 (1.8) | 0.97 (0.92-1.01) | 1.000 | 1.000 |
| GI upset (1), *n* (%) | 0 (0.0) | 1 (1.8) | 0.97 (0.92-1.01) | 1.000 | 1.000 |
| Facial itchiness (1), *n* (%) | 0 (0.0) | 1 (1.8) | 0.97 (0.92-1.01) | 1.000 | 1.000 |
| Floaters (1), *n* (%) | 0 (0.0) | 1 (1.8) | 0.97 (0.92-1.01) | 1.000 | 1.000 |
| Blepharospasm (1), *n* (%) | 0 (0.0) | 1 (1.8) | 0.97 (0.92-1.01) | 1.000 | 1.000 |
| Cough (1), *n* (%) | 0 (0.0) | 1 (1.8) | 0.97 (0.92-1.01) | 1.000 | 1.000 |
| Lower limbs weakness (1), *n* (%) | 1 (50.0) | 0 (0.0) | 0.02 (0.01-0.12) | **0.034** | 0.180 |
